# Imbalance in Bone Morphogenic Proteins 2 and 7 Is Associated with Renal and Cardiovascular Damage in Chronic Kidney Disease

**DOI:** 10.3390/ijms24010040

**Published:** 2022-12-20

**Authors:** Francisco Javier Manzano-Lista, Marta Sanz-Gómez, Daniel González-Moreno, Elena Vega-Martín, Marta Gil-Ortega, Angela Schulz, Miguel Ángel Rubio, Gema Ruiz-Hurtado, Luis Miguel Ruilope, Isabel Aránguez, Reinhold Kreutz, María S. Fernández-Alfonso

**Affiliations:** 1 Instituto Pluridisciplinar, Facultad de Farmacia, Universidad Complutense de Madrid, 28040 Madrid, Spain; 2 Departamento de Ciencias Farmacéuticas y de la Salud, Facultad de Farmacia, Universidad CEU-San Pablo, 28925 Madrid, Spain; 3 Department of Clinical Pharmacology and Toxicology, Charité-Universitätsmedizin, 10117 Berlin, Germany; 4 Servicio de Endocrinología y Nutrición, Hospital Clínico San Carlos, IdISSC, 28040 Madrid, Spain; 5 Unidad de Hipertensión, Instituto de Investigación Imas12, Hospital Universitario 12 de Octubre, 28041 Madrid, Spain; 6 Departamento de Salud Pública y Medicina Preventiva, Universidad Autónoma de Madrid, 28029 Madrid, Spain

**Keywords:** chronic kidney disease, cardiovascular disease, arterial stiffness, bone morphogenetic proteins, Munich Wistar Frömter Rat

## Abstract

Arterial stiffness is a major vascular complication of chronic kidney disease (CKD). The development of renal damage, hypertension, and increased pulse wave velocity (PWV) in CKD might be associated with an imbalance in bone morphogenetic proteins (BMP)-2 and BMP-7. Plasma BMP-2 and BMP-7 were determined by ELISA in CKD patients (stages I–III; n = 95) and Munich Wistar Frömter (MWF) rats. Age-matched Wistar rats were used as a control. The expression of *BMP-2*, *BMP-7*, and profibrotic and calcification factors was determined in kidney and perivascular adipose tissues (PVAT). BMP-2 was higher in stage III CKD patients compared to control subjects. BMP-7 was lower at any CKD stage compared to controls, with a significant further reduction in stage III patients. A similar imbalance was observed in MWF rats together with the increase in systolic (SBP) and diastolic blood pressure (DBP), or pulse wave velocity (PWV). MWF exhibited elevated urinary albumin excretion (UAE) and renal expression of *BMP-2* or kidney damage markers, *Kim-1* and *Ngal*, whereas renal *BMP-7* was significantly lower than in Wistar rats. SBP, DBP, PWV, UAE, and plasma creatinine positively correlated with the plasma BMP-2/BMP-7 ratio. Periaortic and mesenteric PVAT from MWF rats showed an increased expression of *BMP-2* and profibrotic and calcification markers compared to Wistar rats, together with a reduced *BMP-7* expression. BMP-2 and BMP-7 imbalance in plasma, kidney, and PVATs is associated with vascular damage, suggesting a profibrotic/pro-calcifying propensity associated with progressive CKD. Thus, their combined analysis stratified by CKD stages might be of clinical interest to provide information about the degree of renal and vascular damage in CKD.

## 1. Introduction

Chronic kidney disease (CKD) is a progressive condition with high morbidity and mortality and is especially prevalent in the diabetic and hypertensive adult population [1,2]. It is a major public health problem worldwide and will soon become the fifth leading cause of death globally [1]. Patients with CKD exhibit an elevated cardiovascular risk from stages I–III with an exponentially increasing risk of cardiovascular death as kidney function worsens (CKD stages IV–V) [2,3,4].

Increased arterial stiffness is a major and often overlooked vascular complication of CKD leading to increased cardiac workload, reduced coronary artery perfusion, and chronic cardiac dysfunction [5,6]. One cause of reduced arterial elasticity is vascular remodeling, defined as a structural change of the vascular wall due to changes in extracellular matrix composition and cellularity. Another cause is calcification of the vascular wall, both at the intima and media, although medial calcification is more frequent in patients with CKD [7]. These alterations lead to an increase in arterial pulse wave velocity (PWV) [6,8,9], which represents a strong independent predictor of cardiovascular mortality in patients with CKD [5].

Bone morphogenetic proteins (BMPs) are growth factors belonging to the transforming growth factor beta (TGF_β_) superfamily which are involved in a variety of processes not only restricted to bone formation [10]. Hence, BMP-7 is highly expressed in the kidney [11], where it is required for kidney development [12,13]. Levels of BMP-7 are reduced in both experimental models and patients with renal diseases, i.e., diabetic nephropathy, hypertensive nephrosclerosis, pyelonephritis, obstructive uropathy, and acute nephrotoxicity [14,15,16,17,18]. Of interest, treatment with recombinant BMP-7 exerts renal protective effects [19,20] because of a profound reduction in renal fibrosis [21,22].

BMP-2 represents another important BMP with procalcifying properties in CKD [23]. It is expressed in aortic smooth muscle cells [23,24] where it produces calcified nodules similar to those found in bone cell cultures [24]. Plasma BMP-2 levels are increased in CKD patients and negatively correlate with glomerular filtration rates (GFR) [23,25]. In addition, BMP-2 positively correlates with oxidative stress [26], which is increased in CKD patients [27,28]. Although both BMP-7 and BMP-2 changes have been described in established CKD, possible alterations in the onset of CKD are not known and a detailed assessment stratified by CKD stage is lacking.

Against this background, we hypothesize that an imbalance in BMP-2 and BMP-7 occurs at CKD stages I-III associated with the development of hypertension, vascular stiffness, and progressive kidney damage. To test this hypothesis, we analyzed plasma BMP-2 and BMP-7 levels in the Munich Wistar Frömter (MWF) rat, a genetic model with spontaneous nondiabetic albuminuria that mirrors several features observed in patients with CKD [28], including increased oxidative stress, hypertension, alterations in elastin/collagen balance, elevated MMP-9 activity, and arterial stiffness [27,29]. With a translational perspective, we also determined BMP-2 and BMP-7 levels in the plasma of patients with CKD at stages I-III. To determine if plasma BMP-2 and BMP-7 imbalance might reflect organ damage, we determined *BMP-2* and *BMP-7* expression in kidney and perivascular adipose tissue (PVAT) of MWF rats, together with the expression of renal damage markers, *Kim-1* and *Ngal,* as well as profibrotic and procalcifying factors *Runx2*, *Bglap*, *ALP*, *Col1A1,* and *TGF_β_*.

## 2. Results

### 2.1. Demographic, Clinical, and Biochemical Parameters in Study Subjects

Table 1 shows the demographic, clinical, and biochemical characteristics of the subjects included in this study. There was no significant difference in body mass index (BMI) values, total cholesterol, HDL-C, LDL-C, triglycerides, plasma glucose and glycosylated hemoglobin between controls or CKD stages. Almost all CKD patients had a previous diagnosis of hypertension at the time of enrollment. However, systolic (SBP) and diastolic blood pressure (DBP) were similar between groups, indicating a well-controlled hypertension in CKD patients. Markers of renal function, such as creatinine or NGAL, were higher in CKD patients, whereas uric acid levels were not different between groups.

### 2.2. CKD Patients Show an Imbalance in Plasma BMP Levels Associated with 25-OH-Cholecalciferol Deficiency

**Figure 1:**
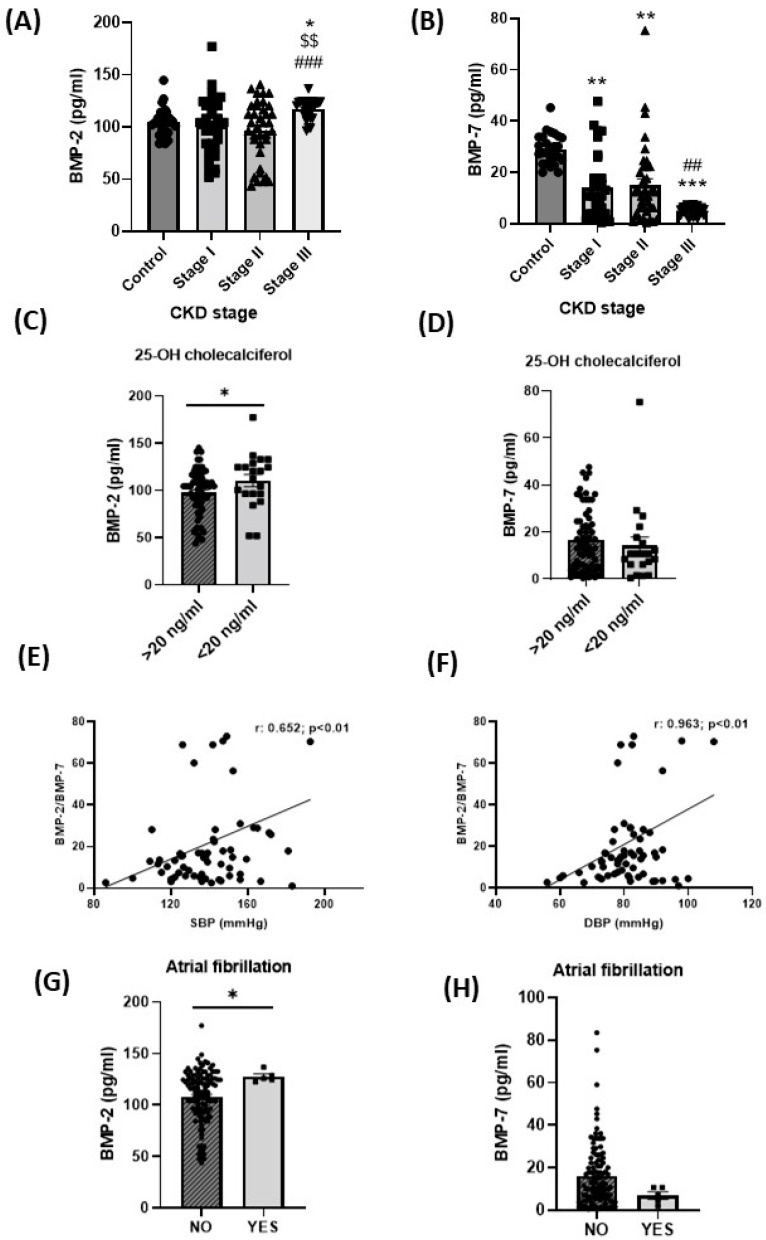
BMP-2 and BMP-7 levels in patients with CKD. Plasma levels of (**A**) BMP-2 and (**B**) BMP-7 in patients with different stages of CKD or healthy controls. Plasma levels of (**C**) BMP-2 and (**D**) BMP-7 according to 25-OH-cholecalciferol deficiency (<20 ng/mL). Correlation between the BMP-2/BMP-7 ratio with (**E**) SBP and (**F**) DBP. Plasma levels of (**G**) BMP-2 and (**H**) BMP-7 according to atrial fibrillation. Data are expressed as the mean ± SEM of n = 26–37. * *p* < 0.05, ** *p* < 0.01, and *** *p* < 0.001 compared to control, $$ *p* < 0.01 compared to stage I, ### *p* < 0.001, ## *p* < 0.01 compared to stage II; * *p* < 0.05 in (**C**,**D**) versus 25-OH-cholecalciferol >20 ng/dL.

Plasma BMP-2 levels were significantly higher in stage III CKD patients as compared to controls, whereas levels were similar to controls in CKD stage I or II patients (Figure 1A). BMP-7 concentration was significantly lower at any stage of CKD compared to the control group and this difference was more pronounced in stage III patients (Figure 1B). Although the mean value of SBP and DBP was not different between groups, there was a positive correlation between SBP or DBP with a BMP-2/BMP-7 ratio (Figure 1E,F).

Since almost 45% of the CKD patients showed 25-OH-cholecalciferol deficiency, we next determined the possible association between it and BMP imbalance. In this group, plasma BMP-2 concentration was higher compared with patients with normal 25-OH-cholecalciferol levels (Figure 1C). No changes in BPM-7 concentrations were detected in CKD patients with 25-OH-cholecalciferol deficiency (Figure 1D). A small subgroup of CKD patients with atrial fibrillation (n = 5) showed a significant BMP-2/BMP-7 imbalance with significantly higher (*p* < 0.05) BMP-2 and lower (*p* < 0.07, non-significant) BMP-7 concentrations compared with CKD patients without atrial fibrillation (Figure 1G,H). The level of 25-OH-cholecalciferol in those patients was of 15.63 ng/mL, *p* < 0.07.

### 2.3. MWF Rats Show an Imbalance in Plasma BMP Levels Associates with 25-OH-Cholecalciferol Deficiency

**Figure 2:**
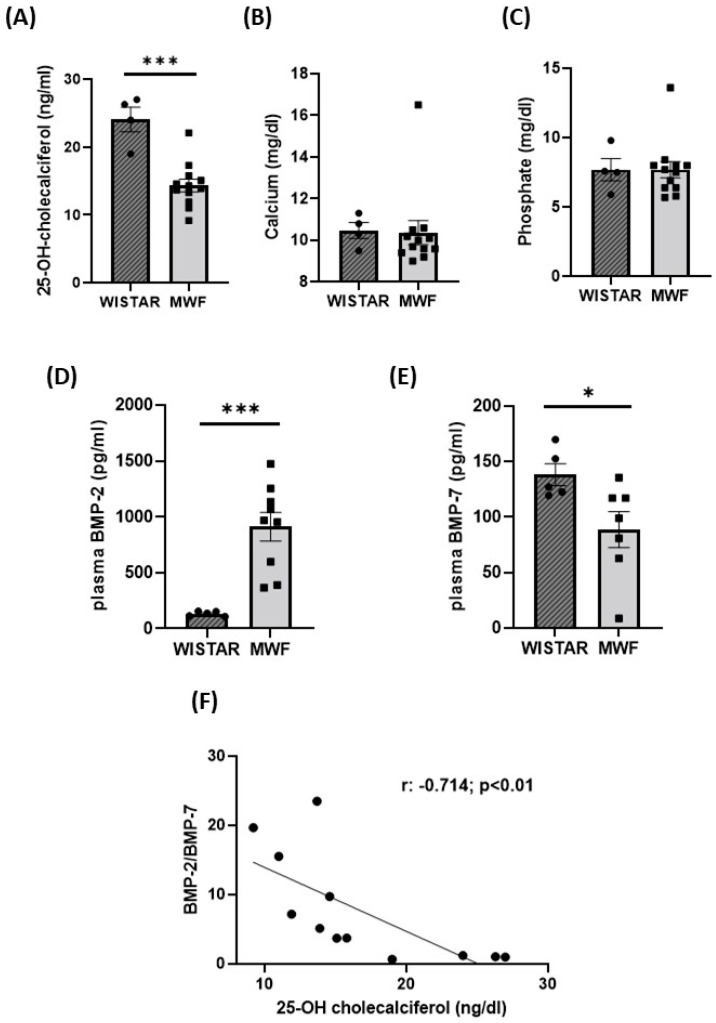
Calcification parameters in Wistar and MWF rats. Plasma levels of (**A**) 25–OH–cholecalciferol, (**B**) calcium, (**C**) phosphate, (**D**) BMP-2, and (**E**) BMP-7. (**F**) Correlation between 25-OH-cholecalciferol and BMP-2/7 ratio. Data are expressed as the mean ± SEM of n = 5–11 animals per group. * *p* < 0.05, and *** *p* < 0.0001 compared to Wistar.

Plasma 25-OH-cholecalciferol levels were significantly lower in MWF than in Wistar rats (Figure 2A). No changes in calcium (Figure 2B) or phosphate (Figure 2C) levels were detected between groups. Plasma BMP-2 concentrations were significantly higher (Figure 2D), whereas BMP-7 was lower in MWF compared to Wistar rats (Figure 2E). Plasma 25-OH-cholecalciferol concentration negatively correlated with the BMP-2/BMP-7 plasma ratio.

### 2.4. MWF Rats Show an Imbalance in Renal BMP Expression Associated to Kidney Damage

**Figure 3:**
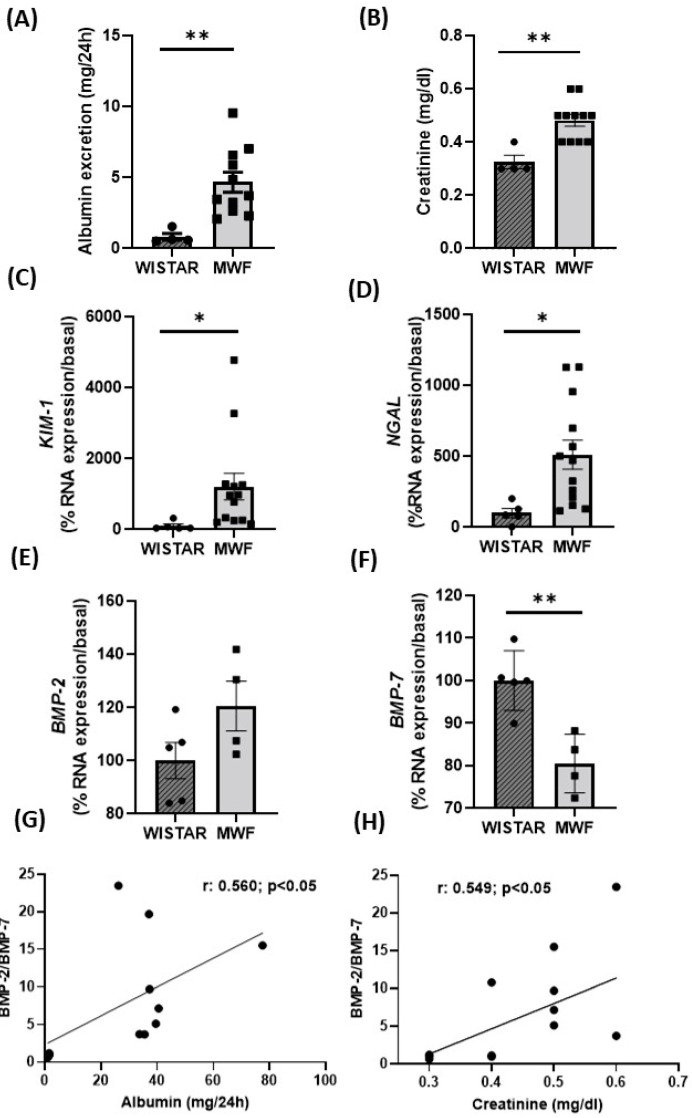
Renal function parameters in Wistar and MWF rats. (**A**) Urinary albumin excretion (UAE) and (**B**) creatinine plasma levels in Wistar and MWF rats. Renal mRNA expression of (**C**) *KIM-1*, (**D**) *NGAL*, (**E**) *BMP-2*, and (**F**) *BMP-7* in Wistar and MWF rats. Correlation between the BMP-2/7 ratio and (**G**) urinary albumin excretion (UAE) or (**H**) creatinine plasma levels. Data are expressed as the mean ± SEM of n = 5–11 animals per group. * *p* < 0.05 and ** *p* < 0.01 compared to Wistar.

Both UAE and plasma creatinine concentrations were higher in MWF compared to Wistar rats (Figure 3A,B). Kidney weight was lower in MWF rats (kidney_Wistar_ = 35 ± 0.4 mg/mm tibia; kidney_MWF_ = 23 ± 0.1 mg/mm tibia). To further assess kidney damage, mRNA levels of tubular damage markers, *Kim-1* and *Ngal*, were determined. Expression of both markers was significantly higher in the MWF compared to the Wistar group (Figure 3C,D, respectively). Renal mRNA expression of *BMP-2* was higher in MWF than in Wistar rats (Figure 3E), whereas *BMP-7* expression was significantly lower (Figure 3F). A positive correlation was detected between the BMP-2/BMP-7 ratio and both UAE (Figure 3G) or plasma creatinine (Figure 3H).

### 2.5. MWF Rats Show an Increase in Blood Pressure and Pulse Wave Velocity Associated with the BMP-2/BMP-7 Imbalance

**Figure 4:**
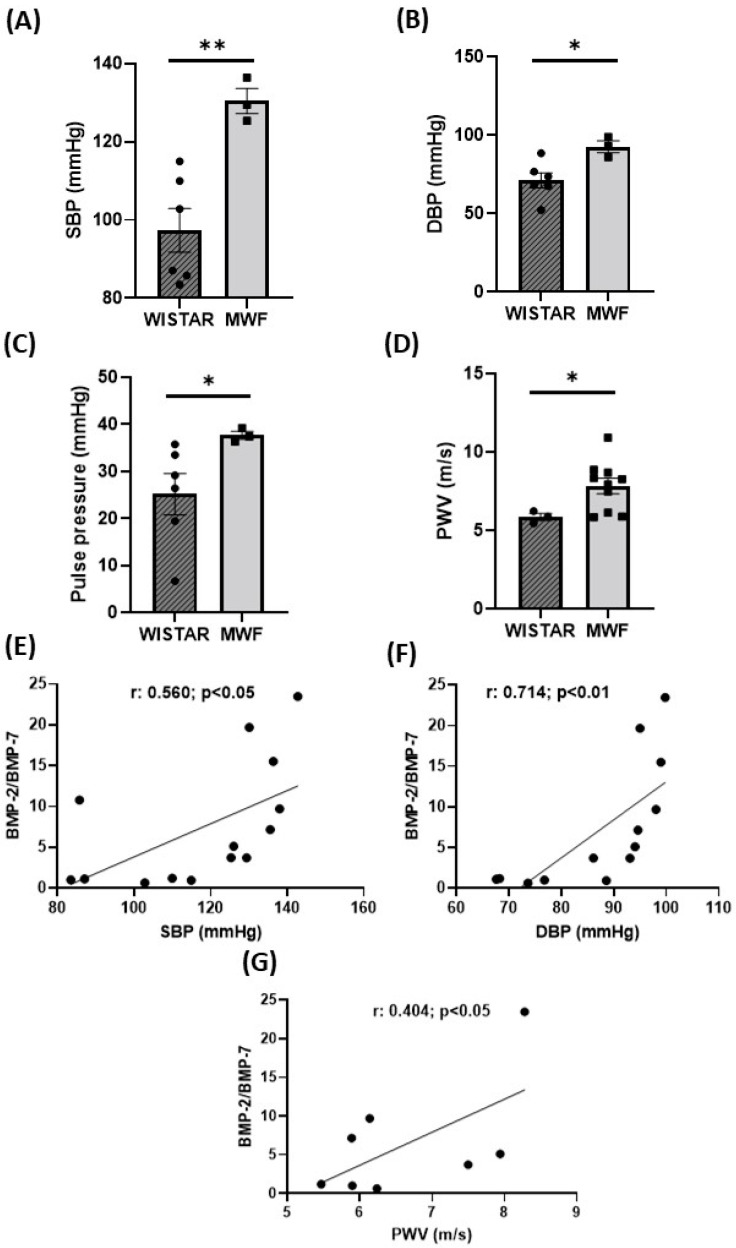
Hemodynamic parameters in Wistar and MWF rats. (**A**) Systolic blood pressure (SBP), (**B**) diastolic blood pressure (DBP), (**C**) pulse pressure (PP), and (**D**) pulse wave velocity (PWV) in Wistar and MWF rats. Correlation between the BMP-2/7 ratio and (**E**) systolic blood pressure (SBP), (**F**) diastolic blood pressure (DBP), or (**G**) pulse wave velocity (PWV). Data are expressed as the mean ± SEM of n = 5–11 animals per group. * *p* < 0.05 and ** *p* < 0.01 compared to Wistar.

The MWF group showed an elevation of both SBP (Figure 4A) and DBP (Figure 4B), as well as increased PP (Figure 4C) and PWV (Figure 4D) compared to the Wistar group. A positive correlation was observed between the BMP-2/BMP-7 ratio and SBP (Figure 4E), DBP (Figure 4F) and PWV (Figure 4G), respectively.

### 2.6. Perivascular Adipose Tissue from MWF Rats Shows an Altered Expression of BMP-2, BMP-7, and Profibrotic and Calcification Markers

**Figure 5:**
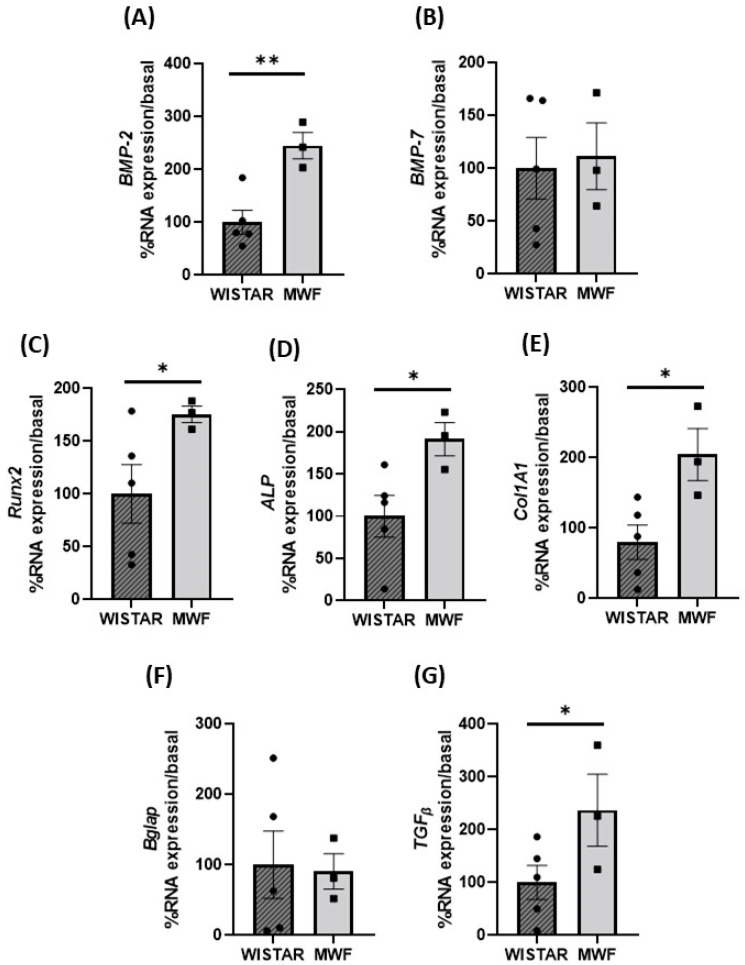
Expression of profibrotic and calcification makers in mesenteric perivascular adipose tissue (PVAT) of Wistar and MWF. mRNA expression level (**A**) *BMP-2*, (**B**) *BMP-7*, (**C**) *Runx2*, (**D**) *Bglap*, (**E**) alkaline phosphatase (*ALP*), (**F**) collagen 1A1 (*Col1A1*), and (**G**) TFG_β_ mRNA expression level. Data are expressed as the mean ± SEM of n = 3–5 animals per group). * *p* < 0.05, and ** *p* < 0.01 compared to Wistar.

We have previously described that the mesenteric arteries of MWF rats have an increased intrinsic arterial stiffness due to alterations in the elastin/collagen balance and increased MMP-9 activity [27,29]. Therefore, we sought to determine the mRNA expression profile of BMPs and calcification and profibrotic markers in mesenteric PVAT that have the characteristics of white adipose tissue. Expression of *BMP-2*, *Runx2*, alkaline phosphatase (*ALP)*, *collagen 1A1*, and *TGF_β_* were significantly higher in mesenteric PVAT than in MWF (Figure 5A,C–E,G, respectively). No differences between groups were observed for *Bglp* and *BMP-7* (Figure 5B,F, respectively).

**Figure 6:**
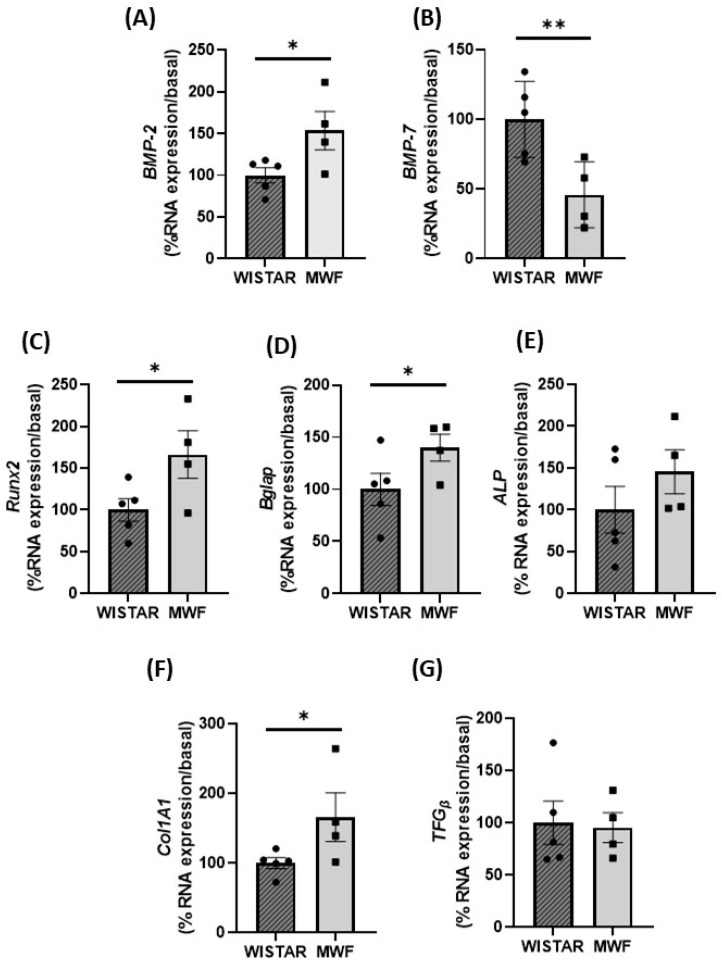
Expression of profibrotic and calcification makers in periaortic adipose tissue (PAT) of Wistar and MWF. mRNA expression level (**A**) *BMP-2*, (**B**) *BMP-7*, (**C**) *Runx2*, (**D**) *Bglap*, (**E**) alkaline phosphatase (*ALP)*, (**F**) Collagen 1A1 (*Col1A1)* and, (**G**) *TFG*_β_ mRNA expression level. Data are expressed as the mean ± SEM of n = 5 animals per group. * *p* < 0.05, and ** *p* < 0.01 compared to Wistar.

To determine whether changes in the expression pattern of these markers also applied to other PVAT depots, we analyzed periaortic AT, which has the characteristics of brown adipose tissue. Expression of *BMP-2, Runx2, Bglap*, and *Collagen 1A1* were significantly higher in the PAT from MWF compared to Wistar (Figure 6A,C,D,F, respectively). *BMP-7* mRNA expression was lower in MWF PAT (Figure 6B) with no changes in alkaline phosphatase (*ALP*) or *TGF_β_* between groups (Figure 6E,G, respectively).

## 3. Discussion

Our study shows an imbalance in BMP-2 and BMP-7 plasma levels in both CKD patients and MWF rats associated with the severity of CKD, 25-OH-cholecalciferol deficiency, and blood pressure. Plasma levels BMP-2 and BMP-7 reflect the expression level of these factors in kidney and PVATs of MWF rats together with increased kidney damage, profibrotic, and calcification factors in these tissues. The correlation between the BMP-2/BMP-7 ratio and kidney function, blood pressure and PWV in the CKD rat model suggests that it is the imbalance between both factors which is decisive, more so than the isolated up- or downregulation of BMP-2 or BMP-7, respectively. Thus, we suggest that the BMP-2/BMP-7 ratio might be of clinical interest to assess early renal and cardiovascular damage in CKD patients.

Previous studies have shown lower BMP-7 levels in several types of kidney damage, both in experimental models [19,20,22,30,31,32] and in patients [14,15,16,17,18]. However, a more detailed assessment of BMP-7 levels stratified by CKD stage is lacking. The herein reported reduction of plasma BMP-7 already in CKD stage I patients suggests that the decrease in BMP-7 might be an early event in CKD development, reflecting a reduction in renal tubular BMP-7 expression. In this line, Wong et al. suggested that decreased BMP-7 levels in patients with type 2 diabetes, who subsequently develop major renal end points, might predict kidney end points much more strongly than the best currently available risk marker [33]. In fact, a loss of viable renal mass correlates with lower BMP-7 plasma values [34], as we also observe in MWF kidneys, which are smaller and exhibit a genetic nephron deficit [35].

BMP-2 is a well-known procalcifying factor. It is upregulated in human atherosclerotic plaques [24] and in calcified aortic stenosis [36] and produces calcified nodules in cultured vascular smooth muscle cells [24]. BMP-2 levels are higher in CKD patients before dialysis [25], as well as in uremic serum from haemodialysis patients without residual renal function [25]. These levels inversely correlate with eGFR in the CKD population [23], although stratification by stage has not been analyzed. The herein reported increase in BMP-2 plasma levels at stage III CKD patients, but not at earlier stages, might reflect the turning point in vascular calcification, taking into consideration that its prevalence approximately doubles from 25% at CKD stage III [37] to 50% at stage V [38]. Interestingly, Vit D deficiency, which is clearly linked to arterial stiffness, vascular calcification, and CV mortality in CKD [39], is associated with increased BMP-2 levels both in our CKD patient cohort and rat model. Moreover, a highly significant increase of BMP-2 was observed in a small subgroup of CKD patients with atrial fibrillation and 25-OH-cholecalciferol deficiency. 25-OH-cholecalciferol stimulates sclerostin and inhibits BMP2 production, thus mitigating osteoblastic transdifferentiation and calcification of soft tissues in a murine CKD model [40]. However, possible valve calcification in the atrial fibrillation patient subgroup cannot be excluded. Further studies with higher patient numbers and defined patient cohorts will be necessary to address this question.

CKD patients exhibit increased oxidative stress [27,28], which seems to play a far more important role in vascular disease in these patients than in the general population [41]. Since oxidative stress stimulates the pro-calcifying effects of BMP-2 in human endothelial cells [36], it is likely that the increase in BMP-2 contributes to an impaired endothelial function. Previous data from our group show that impaired endothelial function in the MWF rat is linked to an increase in oxidative stress by an abnormal formation of reactive oxygen species, both in the vascular endothelium and in the kidney [42], rather than to a decrease in nitric oxide synthesis [43], supporting the primary causative role of endothelial dysfunction in renal impairment [42].

BMP-7 is a physiologic antagonist of TFG_β_, an essential bystander in renal fibrosis as a central event in the progression of CKD [44,45]. Therefore, the renal protective functions of BMP-7 have been attributed to its antifibrotic properties [22,46,47,48,49], through the suppression of myofibroblast activation and the inhibition of collagen and fibronectin synthesis [49]. Antifibrotic effects of BMP-7 have been shown in extrarenal tissues such as the liver [50,51] and the heart [52,53]. Interestingly, *BMP-7* expression is reduced in periaortic AT and mesenteric PVAT in MWF rats and is associated with an increase in TFG_β_ and collagen expression. In parallel, there is an increase in *BMP-2* expression in both PVATs together with an upregulation of the osteogenic and calcification factors *Runx2, ALP*, and *osteocalcin (Bglap)* [54,55,56,57]. This is the first report describing the expression of these osteogenic factors in both white mesenteric PVAT and brown periaortic AT. Moreover, phenotypic changes in PVAT during CKD development have not been addressed until now. PVAT is a source of numerous vasoactive factors with paracrine effects on both vascular function and structure [58,59]. In pathophysiological situations, such as hypertension, diabetes, or obesity, changes in the expression pattern of some vasoactive factors induce PVAT dysfunction, thus creating a pro-oxidant, proinflammatory, contractile, and trophic environment that leads to vascular remodeling and arterial stiffness [58,59]. In this scenario, the imbalance between BMP-2 and BMP-7, together with the profibrotic and calcification marker expression in PVAT, might have a paracrine effect, leading to vascular dysfunction, an increase in systolic and diastolic blood pressure, and to higher PWV. The imbalance between BMP-2 and BMP-7 in the kidney is reflected as albuminuria, supporting the link between vascular and renal dysfunction [24,29,42]. The significant correlation between the BMP-2/BMP-7 ratio and both systolic and diastolic blood pressure in CKD patients supports this possibility.

Several limitations of this study are important to note. First, PWV was not determined in the CKD patient cohort. Although the MWF rat is a good experimental model that mirrors many features observed in patients with CKD, assessment of PWV in CKD patients at different CKD stages will be of interest. Second, the patient number in each CKD stage is too small to be able to perform homogenous subgroups, i.e., diabetic or hyperlipidemic patients, and a greater CKD patient cohort will be necessary for further studies. Third, the younger age of the control group might carry a potential bias in the data interpretation as the average age between the different CKD groups increases with the CKD stage and BMP-7 levels seem to be higher at younger ages, at least in some diabetic mice models [60]. Fourth, this is an association study and further work will be needed to determine if the imbalance in the BMP-2/BMP-7 ratio is causal and if it might add predictive value to CV damage in CKD patients. Nevertheless, the novelty of our study is to propose the combined analysis of BMP-2 and BMP-7 in CKD and to stratify it by stages. Since the BMP-2/BMP-7 ratio in MWF rats correlates with (i) 25-OH-cholecalciferol deficiency, (ii) renal function worsening, and (iii) SBP, DBP, and PWV, we propose that its determination might be of clinical interest and could provide information about the degree of renal and vascular damage in CKD.

In conclusion, an imbalance in BMP-2 and BMP-7 levels observed in plasma, kidney, and PVATs is associated with 25-OH-cholecalciferol deficiency, increases in blood pressure and arterial stiffness, and might indicate a profibrotic/pro-calcifying propensity associated with progressive CKD. Thus, we suggest that the BMP-2/BMP-7 ratio might be of clinical interest once validated in a higher number of patients. Strategies to reduce and/or correct this imbalance in experimental models and clinical practice are warranted.

## 4. Materials and Methods

### 4.1. Study Population

Plasma samples, anticoagulated with ethylenediamine tetraacetic acid (EDTA), were obtained from 121 CKD patients (>18 years old) from the Hypertension Unit of the Nephrology Department of the Hospital Universitario 12 de Octubre in Madrid. Patients were classified into three groups according to the estimated GFR (eGFR), calculated using the CKD-EPI formula [61]: (i) >90 mL/min/1.73 m^2^; (ii) 90 to 60 mL/min/1.73 m^2^; and (iii) 59 to 30 mL/min/1.73 m^2^. Patients with diabetes or with eGFR < 30 mL/min/1.73 m^2^ on hemodialysis were excluded from the study. Control group were healthy subjects (n = 26) with normal weight and without hypertension.

Clinical history data were used to obtain anthropometric measurements (body mass index, BMI), systolic (SBP) and diastolic blood pressure (DBP) values, glycemic parameters (plasma glucose and glycated hemoglobin), lipid profile (cholesterol, triglycerides, HDL-C and LDL-C), and renal function parameters (proteinuria, plasma albumin, and creatinine). A diagnosis of essential hypertension was based on a previous history of hypertension or BP/DBP ≥ 140/90 mmHg measured in the clinic following the European guidelines [62]. Deficiency in 25-OH-cholecalciferol (<20 ng/mL) was considered according to the clinical practice guidelines of the Endocrine Society [63]. All patients signed an informed consent form prior to the inclusion in the study which was approved by the Ethics Committee of the hospital and conducted in accordance with the principles of the Declaration of Helsinki.

### 4.2. Animals

Twenty-two-week-old male normotensive and normoalbuminuric Wistar (W; control group; Charles River, Barcelona, Spain; n = 5) and MWF rats (CKD group; Charité–University Medicine Berlin, Germany; n = 5) were housed in groups of two under controlled dark–light cycles (12 h/12 h), temperature conditions, and with access to food (A.04, Panlab, Barcelona, Spain) and water ad libitum. Urinary albumin excretion (UAE) was determined in 24 h-old urine collected in metabolic cages after 24 h acclimation as previously described [27]. On the last day, both carotid and femoral arteries were catheterized under anesthesia (80 mg/kg ketamine hydrochloride and 12 mg/kg xylazine hydrochloride, i.p.) and blood pressure waves were recorded in a PowerLab system (ADInstruments, Oxford, UK) to obtain SBP, DBP, and pulse pressure (PP) [22]. PWV, which represents the pressure waveform traveling along the aorta and large arteries during each cardiac cycle, was calculated applying the following formula: D (meters)/Δt (seconds). D is the distance between the two arteries, whereas Δt is the time delay between the carotid and the femoral pressure waves [64].

After hemodynamic measurements, animals were euthanized by anesthesia overdose, blood was collected in heparinized tubes to obtain plasma, and tissues (kidney, mesenteric PVAT and periaortic AT) were removed and frozen for further study (plasma determinations and PCR studies). All experimental procedures were performed in accordance with the European Union Laboratory Animal Care Rules (86/609/ECC directive) and were approved by the Animal Research Committee of Complutense University (PROEX 205/18). All efforts were made to avoid animal suffering in accordance with the ARRIVE guidelines for reporting experiments involving animals.

### 4.3. Plasma Determinations in Patients and Rats

Measurements of potassium, calcium, phosphate, 25-OH-cholecalciferol and creatinine were performed by Megalab^®^ laboratory on a plasma sample. Determination of total plasma BMP-2 and BMP-7 concentration in both patients and rats was performed using specific ELISA kits according to the manufacturer’s specifications (Quantikine^®^, R&D Systems Inc., Minneapolis, MN, USA). Rat and human BMP-2 shares 100% homology (https://www.ncbi.nlm.nih.gov/homologene/926 (accessed on 13 September 2022)), whereas BMP-7 shares 97% homology (https://www.ncbi.nlm.nih.gov/homologene/20410 (accessed on 13 September 2022)). The ELISA for BMP-2 (DBP-200) has a sensitivity of 29 pg/mL and an intra- and interassay coefficient of variation of 2.6% and 7.3%, respectively. The ELISA for BMP-7 (NBP2-70002) has a sensitivity of 9.38 pg/mL and an intra- and inter-assay coefficient of variation of 6.77% and 6.60%, respectively. The ELISA for NGAL (DLCN20, Quantikine^®^, R&D Systems Inc., Minneapolis, MN, USA) has a sensitivity of 0.04 ng/mL and an intra- and inter-assay coefficient of variation of 3.6% and 7.9%, respectively.

### 4.4. RNA Extraction and Real-Time PCR (RT-qPCR)

Total RNA was isolated from the W and MWF kidney and PVAT samples (n = 5/strain for each tissue) using Qiazol Reagent (Qiagen, Düsseldorf, Germany). The samples were processed with 500 µL trizole using 35–45 mg of tissue. After centrifugation (12,000 rpm) and 200 µL chloroform addition, centrifugation (12,000 rpm) was repeated. The supernatant was collected, mixed with 150 µL isopropanol, and centrifuged (12,000 rpm). The pellet was resuspended in 300 µL 75% ethanol. Afterwards, the instructions of the RNA Spin illustraTM kit (GE Healthcare, Chicago, IL, USA) were followed and the concentration and purity of RNA were assessed with NanoDropTM 2000/c (Fisher Scientific, Pittsburgh, PA, USA). Reverse transcription was performed on 500 ng of RNA with iScript cDNA synthesis kit (BioRad, Hercules, CA, USA) using random hexamer primers (Table 2) for *Kim-1* and *Ngal* (kidney) or *Runx2*, *Bglap*, *ALP*, *Col1A1*, *TGF_β_*, (PVAT), *BMP-2,* and *BMP-7* (kidney and PVAT). Both *Kim-1* and *Ngal* are markers for kidney injury, specifically for tubular damage, and are a positive control of CKD in the MWF rat. Optimal annealing temperature and amplicon sizes were checked for each pair of primers. RT-qPCR analyses were performed in a CFX96 Instrument (BioRad, Hercules, CA, USA). A total of 10 ng of cDNA from ten samples of each group were run in duplicate. mRNA levels were determined using intron-skipping primers, GADPH and ATPF-1 as housekeeping genes for normalization, and SYBR Green Master Mix (Applied Biosystems, Foster City, CA, USA).

### 4.5. Statistical Analysis

Based on previous studies, the number of animals per group was n = 5, accepting an alpha risk of 0.05 and a beta risk of 0.2 in a bilateral contrast, to detect a difference equal to or greater than 1.6 units. A standard deviation of 1.2 and a loss-to-follow-up rate of 10% were assumed. Continuous variables were compared by Student’s *t* test or one-way ANOVA with the Newman–Keuls test, and nonparametric variables were compared by the Kruskal–Wallis test. Categorical variables were compared using Fisher’s exact test. Correlations were calculated using Pearson’s correlation coefficient. Data analysis was performed with SPSS v26 software for patient data and with GraphPad Prism 8 for animal data. Data are presented as mean ± standard error of the mean and statistical significance was set at *p* < 0.05.

## Figures and Tables

**Table 1:** Demographic parameters.

	Control (n = 26)	Stage I (n = 32)	Stage II (n = 37)	Stage III (n = 26)
Number of female (%)	18 (69.2%)	13 (40.6%)	15 (40.5%)	10 (38.5%)
Age (18–65 years) (%)	26 (100.0%)	23 (71.9%)	16 (43.2%)	2 (7.7%)
Age (years)	46.4 ± 7.3	57.8 ± 8.2	64.9 ± 8.8	72.0 ± 10.1
**Kidney function markers**				
Creatinine (mg/dL)	0.77 ± 0.2	0.78 ± 0.1	0.97 ± 0.1 *	1.57 ± 0.6 *#
eGFR (ml/min/1.73 m^2^)	113.7 ± 6.1	95.25 ± 5.2	74.08 ± 9.0 *	44.50 ± 13.4 *
Proteinuria (>30 mg/dL). (N/%)	--	9 (28.1%)	25 (67.7%)	6 (23.1%)
Albumin (mg/dL)	--	4.56 ± 0.8	4.55 ± 0.7	4.36 ± 0.6
Albumin/creatinine	--	26.34 ± 35.8	39.07 ± 58.9	45.4 ± 70.1
NGAL (ng/mL)	--	43.09 ± 27.35	60.08 ± 39.01	142.87 ± 160.1 ***##
Uric acid (mg/dL)	5.36 ± 1.1	5.55 ± 1.4	6.11 ± 1.8	6.92 ± 2.0
**Anthropometric measurements**				
BMI (18.5–24.9 kg/m^2^)	24 (92.3%)	4 (12.5%)	8 (21.6%)	11 (42.3%)
BMI (25–29.9 kg/m^2^)	2 (7.7%)	13 (40.6%)	12 (32.4%)	8 (30.8%)
BMI (>30 kg/m^2^)	0 (0.0%)	15 (46.9%)	17 (45.9%)	7 (26.9%)
BMI (kg/m^2^)	24.21 ± 6.8	30.15 ± 4.5	30.38 ± 5.0	30.27 ± 5.9
**Metabolic indicators**				
Blood glucose (mg/dL)	98.23 ± 4.3	111.37 ± 19.0	115.81 ± 25.9	113.19 ± 21.5
Hb1AC (%)	5.1 ± 0.2	5.80 ± 0.6	6.10 ± 0.7	5.81 ± 0.7
Cholesterol (mg/dL)	191.33 ± 21.7	184.75 ± 35.7	171.08 ± 33.5	155.20 ± 39.0
Triglycerides (mg/dL)	117.93 ± 18.7	114.56 ± 39.1	115.97 ± 37.7	130.00 ± 62.5
HDL (mg/dL)	60.61 ± 15.6	55.63 ± 15.6	51.46 ± 12.2	48.01 ± 14.7
LDL (mg/dL)	143.24 ± 14.2	106.18 ± 33.2	96.32 ± 26.9	78.50 ± 31.9
**Cardiovascular parameters**				
Arterial hypertension. n (%)	0 (0.0%)	32 (100.0%)	37 (100.0%)	25 (96.2%)
SBP (mmHg)	127.33 ± 11.3	137.70 ± 14.8	140.29 ± 19.0	139.23 ± 22.4
DBP (mmHg)	74.09 ± 10.6	83.09 ± 9.2	81.15 ± 9.1	82.30 ± 9.0

eGFR (estimated glomerular filtration rate); systolic (SBP) and diastolic blood pressure (DBP) * *p* < 0.05 vs. stage I; *** *p* < 0.001 vs. stage I; # *p* < 0.05 vs. stage II; ## *p* < 0.01 vs. stage II.

**Table 2:** Primer sequences.

Gene	Accession Number	Forward (5′-3′)	Reverse (5′-3′)
Rn *Kim-1*	NM_173149.2	ATTGTTGCCGAGTGGAGAT	TGTGGTTGTGGGTCTTGTAGT
Rn *Ngal*	NM_130741.1	GGCCGACACTGACTACGACC	GCCCCTTGGTTCTTCCGTAC
Rn *BMP-2*	NM_017178.2	CCCCTATATGCTCGACCTGTACC	TGAAAGTTCCTCGATGGCTTCT
Rn *BMP-7*	NM_001191856.2	GAGGGCTGGTTGGTATTTGACA	AACTTGGGGTTGATGCTCTGC
Rn *Runx2*	NM_001278484.2	CACCGTGTCAGCAAAACTTCTTT	CTACGTCGCTCATCTTGC
Rn *ALP*	NM_013059.2	ATGCACAACATCAAGGACATCG	CATCAGTTCTGTTCTTGGGGTACAT
Rn *Bglap*	NM_013414.1	GCTACCTCAACAATGGACTTGGA	GAGCTCACACACCTCCCTGTG
Rn *Col1A1*	NM_053304.1	GGATGCCATCAAGGTCTACTGC	TGAGTGGGGAACACACAGGTCT
Rn *GAPH*	NM_017008.4	AAGGCTGAGAAATGGGAAGCTC	CCATTTGATGTTAGCGGGATCT
Rn *ATPAF-1*	NM_001107959.1	GATCTCTCCAAGAAGCTGCAAG	AAGATGACCCCAAGGCATTTTT

## Data Availability

The data supporting the findings of this study are available from the corresponding author upon reasonable request.
